# Magnetic resonance imaging of the Lisfranc ligament

**DOI:** 10.1186/s13018-018-0968-x

**Published:** 2018-11-12

**Authors:** A. Ablimit, Hui-Yong Ding, Li-Guo Liu

**Affiliations:** 1 Department of orthopedics, Second Affiliated Hospital of Xinjiang Medical University, Urumqi, 830000 China; 2 People’s Hospital of Rizhao, Xinjiang Medical University, No. 136 of Tai’an Road, Rizhao, 276800 Shandong Province China

**Keywords:** The Lisfranc ligament, Lisfranc, Magnetic resonance imaging, Oblique coronal

## Abstract

**Background:**

The Lisfranc joint has complex structures, and articular surfaces overlap on conventional X-ray radiographs. Hence, there is no available auxiliary examination for diagnosing related injuries. At present, few studies on the imaging of Lisfranc ligaments have been reported, and related imaging data are rare. Therefore, no imaging reference can be used for related diagnosis and repair operations. This study aims to observe and describe the morphology and structure of Lisfranc ligaments using magnetic resonance imaging (MRI), in order to provide imaging reference for the diagnosis and repair of Lisfranc joint injuries.

**Methods:**

MRI scanning was performed on 60 sides of normal feet of 30 healthy adult volunteers. In the MRI scanning on the Lisfranc joint, sagittal scanning was focused on the area between the lateral margin and medial margin of the Lisfranc joint, while oblique coronal scanning was focused on the area parallel to the Lisfranc joint clearance. After acquisition of MRI images, data were burned into a CD, and the morphology and structure of the Lisfranc ligament on the MRI image were observed and described. Hence, the imaging parameters of the Lisfranc ligament were acquired, providing an imaging reference for the diagnosis and repair of Lisfranc joint injuries.

**Results:**

By observing the obtained images of the Lisfranc ligament through appropriate MRI scanning, it was found that the Lisfranc ligament originates at the site 12.63 ± 1.20 mm from the lateral side of the base of the medial cuneiform bone, with a length of 8.02 ± 1.5 mm, a width of 2.53 ± 0.61 mm, a height of 6.96 ± 1.01 mm, forms an included angle of 46.79 ± 3.47° with the long axis of the first metatarsal bone, and finally ends at the base of the second phalanx. Detailed imaging parameters of the Lisfranc joint and ligament were obtained from the present imaging experiment, providing an imaging reference for the diagnosis and repair of Lisfranc joint injuries.

**Conclusions:**

On the MRI images, the sagittal section can clearly display the corresponding situation of the Lisfranc joint bone and longitudinal arch of the foot, tolerably display the Lisfranc joint dorsal ligaments and metatarsal ligaments, and poorly display the Lisfranc ligament. The oblique coronal section can clearly display the transverse arch of the foot and clearly display the cross-section of the Lisfranc ligament. The oblique crosssection can clearly display the horizontal arch of the Lisfranc joint and more clearly display its surrounding ligaments and tendons, especially the entire Lisfranc ligament and its attachment points. This is an important section for the diagnosis of Lisfranc ligament injuries. This study provides a certain imaging reference for the MRI scanning, diagnosis, and repair of Lisfranc joint injuries. Further research with large sample size is still needed to confirm the conclusions.

## Background

The Lisfranc joint is an important part of the transverse arch and longitudinal arch of the foot [1]. Its shape can be seen as a wedge surrounded and attached by strong ligaments, allowing it to have a small range of motion [2–8]. The Lisfranc joint plays an important role in the process of walking with the lower extremities, and the so-called Lisfranc ligament is the ligament that originates from the lateral side of the medial cuneiform bone and ends at the medial side of the base of the second metatarsal bone. Since the base of the first and second metatarsal bones lack the adhesion of intermetatarsal ligaments, the Lisfranc ligament plays an important role in maintaining the stability of the medial column and axial column of the foot arch. Lisfranc injuries refer to injuries of the bones, joints, and ligaments of the Lisfranc joint, which are rare in clinical practice; accounting for approximately 0.2% of all fracture cases [9]. Its incidence is higher in male cases and is 2–3 times of that in female cases [10]. The main causes of injury are high-energy damage caused by traffic accidents and relatively low-energy damage caused by high falls [11].

The anatomical structure of the Lisfranc joint is complex, and the sensitivity of the X-ray film is only 84.4% [12]. Therefore, misdiagnosis and missed diagnosis easily occur. Injury diagnosis of the ligaments of the Lisfranc joint is more difficult, particularly for the diagnosis of the Lisfranc ligament (interosseous ligament). The reason is that the location of the Lisfranc ligament is deep, and its length is short; hence, its injury is more difficult to diagnose. Misdiagnosis, missed diagnosis, and untimely or improper treatment often leads to the instability of the Lisfranc joint or even the formation of traumatic arthritis of the Lisfranc joint. With the advent and development of magnetic resonance imaging (MRI) auxiliary diagnosis technology, many scholars have conducted imaging studies on Lisfranc joint injuries using MRI. The image obtained can clearly show the image of the ligament, providing a reliable basis for auxiliary diagnosis [13]. Since the Lisfranc ligament distributes obliquely, its display is generally poor. Based on the results of previous tests, we positioned and scanned the Lisfranc joint from the oblique cross-section parallel to the dorsal foot and oblique coronal-section parallel to the Lisfranc joint clearance, which can be just right to display the entire Lisfranc ligament and attachment points.

Recently, few researches on the imaging of Lisfranc ligaments have been reported, and related imaging data are rare and no imaging reference can be used for the related diagnosis and repair operation of this tissue. Therefore, we conducted this study to analyze the MRI images of the Lisfranc joint in order to provide an imaging basis for the image recognition and damage diagnosis of this ligament.

## Experimental Methods

### Selection of subjects

This study was conducted in accordance with the declaration of Helsinki. This study was conducted with approval from the Ethics Committee of Second Affiliated Hospital of Xinjiang Medical University. Written informed consent was obtained from all participants.

A total of 30 adult volunteers were enrolled. Among these subjects, 16 were male and 14 were female, and the age of these subjects ranged within 22–34 years old, with an average age of 26 years old. Each of the volunteers was examined and determined without deformity and foot trauma, or history of surgery and diseases that may have an impact on the results such as gout, rheumatoid, and diabetes were excluded.

### Scanner and scanning method

An Intera Achieva 1.5-T magnetic resonance machine (PHILIPS, Holland) was used, which was equipped with a high-resolution knee coil. Position and placement: the toe of the subject was first entered, with the planta touching the bed. The foot was placed in the knee coil, and sandbags were placed around the foot for fixation. Scan methods are as follows: (1) the Lisfranc joint was placed in the horizontal lateral position within the coil, adjusted close to the natural state of the body to the maximum extent, and proper fixation was provided. The bit line should regard the fifth metatarsal base as the reference point. (2) The scan axis was perpendicular to the long axis of the calcaneus and foot (sagittal section), which was parallel to dorsal foot (oblique cross-section) and parallel to the Lisfranc joint surface (oblique coronal-section), respectively. (3) Main parameters: T1-vibe was set as the T1 contrast sequence, and FLASH was set as the T2 check sequence. Specific scan parameters were as follows: SE/T1 WI sequence: slice thickness was 3 mm; slice gap was 3 mm; TR was 550 ms and TE was 16 ms; visual field was FOV 180 × 180 mm; matrix was 436 × 512; and acquisition time was 3 min and 35 s. SE/T2 WI sequence: slice thickness was 3 mm; slice gap was 3 mm; TR was 3200 ms and TE was 36 ms; matrix was 512 × 512; visual field was FOV 160 × 160; and acquisition time was 3 min and 54 s. (4) The resulting images were burned on a disc for storage.

### Statistical methodology

In our study, we used the software program SPSS 20.0 to conduct the statistical analysis. All continuous variables were expressed as mean ± standard deviation.

## Results

### Analysis steps

On the MRI images of the sagittal section, oblique coronal section and oblique transverse section, the bone, joint, ligaments and muscles, and other soft tissues of the Lisfranc joint were analyzed, and the MRI images of the Lisfranc joint of 60 sides of feet were measured and analyzed. Image analysis was performed by three physicians who had a chief physician title. The analyzed contents included the Lisfranc joint bone structure display, articular cartilage display, the display of the profile of the ligaments and muscles and attachment points, and the joint space display; the measurement and statistical analysis of imaging parameters of the Lisfranc ligament.

### Lisfranc ligament magnetic resonance imaging measurement data

The outcomes showed that the Lisfranc ligament originates at the site 12.63 ± 1.20 mm from the lateral side of the base of the medial cuneiform bone, with a length of 8.02 ± 1.5 mm, a width of 2.53 ± 0.61 mm, a height of 6.96 ± 1.01 mm, forms an included angle of 46.79 ± 3.47° with the long axis of the first metatarsal bone, and finally ends at the base of the second phalanx (Table 1).

**Table 1:** Lisfranc Ligament magnetic resonance imaging measurement data

Project	*x* ± *s*
Lisfranc distance of ligament starting point to entocuneiform base (mm)	12.63 ± 1.20
Lisfranc ligament length (mm)	8.02 ± 1.50
Lisfranc Ligament width (mm)	2.53 ± 0.61
Lisfranc ligament height (mm)	6.96 ± 1.01
Lisfranc angle between the Lisfranc ligament and the long axis of the first metatarsal bone(°)	46.79 ± 3.47

### The MRI image

**Fig. 1:**
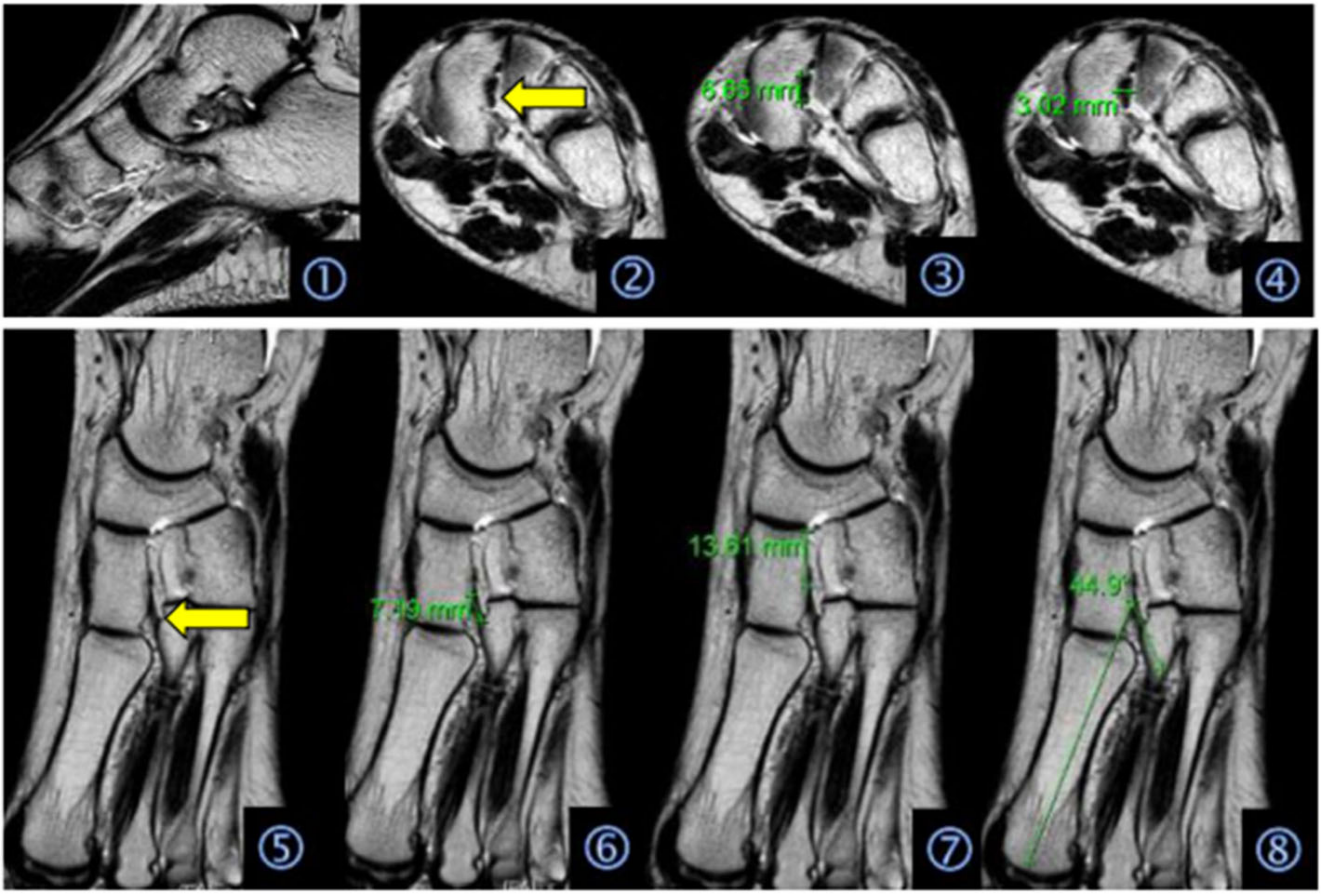
Graphic interpretations: (1) MRI scanning images of the sagittal section; (2) MRI scanning images of the oblique transverse section, where the arrow indicates the Lisfranc ligament; (3) height measurement of the Lisfranc ligament; (4) width measurement of the Lisfranc ligament; (5) MRI images of the oblique coronal section, where the arrow indicates the Lisfranc ligament; (6) length of the measurement of the Lisfranc ligament; (7) measurement of the distance between the origin of the Lisfranc ligament and the base of the medial cuneiform bone; and (8) measurement of the included angle between the Lisfranc ligament and the long axis of the first metatarsal bone

On the MRI images, the sagittal section can clearly display the corresponding situation of the Lisfranc joint bone and longitudinal arch of the foot, tolerably display the Lisfranc joint dorsal ligaments and metatarsal ligaments, and poorly display the Lisfranc ligament. The oblique coronal section can clearly display the transverse arch of the foot and clearly display the cross-section of the Lisfranc ligament. The oblique cross-section can clearly display the horizontal arch of the Lisfranc joint and more clearly display its surrounding ligaments and tendons, especially the entire Lisfranc ligament and its attachment points (Fig. 1).

## Discussion

The outcomes of our study showed that the sagittal section can clearly display the corresponding situation of the Lisfranc joint bone and longitudinal arch of the foot, tolerably display the Lisfranc joint dorsal ligaments and metatarsal ligaments, and poorly display the Lisfranc ligament. The oblique coronal section can clearly display the transverse arch of the foot and clearly display the cross-section of the Lisfranc ligament. The oblique cross-section can clearly display the horizontal arch of the Lisfranc joint and more clearly display its surrounding ligaments and tendons, especially the entire Lisfranc ligament and its attachment points. This is an important section for the diagnosis of Lisfranc ligament injuries. It can be summarized that the Lisfranc ligament originates at the site 12.63 ± 1.20 mm from the lateral side of the base of the medial cuneiform bone, has a length of 8.02 ± 1.5 mm, a width of 2.53 ± 0.61 mm, a height of 6.96 ± 1.01 mm, forms an included angle of 46.79 ± 3.47° with the long axis of the first metatarsal bone, and finally ends at the base of the second phalanx. This study provides a certain imaging reference for the MRI scanning, diagnosis, and repair of Lisfranc joint injuries.

The Lisfranc joint is an important component of the foot arch structure. Its structure is of great significance for the weight-bearing function of the foot. It consists of three parts: the medial column, which consists of the first cuneiform and first metatarsal base; the axial column, which consists of the second and third metatarsal bones and the second and third metatarsal bases. Among these, the second metatarsal bone and third cuneiform form a mortise and tenon structure. This structure is also an important part in maintaining Lisfranc joint stability. Previous studies have emphasized the importance of the third metatarsal bone [14–16]. In recent years, the number of traffic accidents and falling injuries has increased, and Lisfranc joint injuries have also significantly increased. Routine non-enhanced foot X-ray films cannot meet the requirements of the diagnosis of Lisfranc joints, especially for slight Lisfranc joint subluxation, causing the missed diagnosis rate to be as high as 10–20% [17]. Advanced spiral CT post-processing technique can comprehensively observe fractures and dislocations of the midfoot. In particular, three-dimensional reconstruction technology has great advantages in the diagnosis of micro fractures and small dislocations of the foot. Despite all these, non-enhanced X-ray films and CTs have significant inadequate display effects on the injuries of soft tissues, such as ligaments and tendons. MRI has a good ability to distinguish tissues and clearly display the bone, articular cartilage, surrounding ligaments, tendons, and muscle tissues of the Lisfranc joint. Therefore, it is of great significance to read the MRI images of the Lisfranc joint in detail, in order to obtain data for auxiliary diagnosis.

Recently, Thierfelder et al. [18] reviewed the anatomy for each ligament complex or tendon, followed by relevant facts on biomechanics and typical findings in case of injury and confirmed that magnetic resonance imaging (MRI) is invaluable regarding the correct assessment of (partial) ruptures, as well as for evaluating accompanying injuries. Tafur et al. [19] reported the MR imaging features of common osseous, tendon, and ligament abnormalities that affect the midfoot and also presented that MRI plays an important role in the early diagnosis of Lisfranc Ligament. The results of our study provides a certain imaging reference for the MRI scanning, diagnosis, and repair of Lisfranc joint injuries which extended those former studies.

### Limitations

Firstly, the sample size of our study was limited. However, this preliminary research had paved the way for the further research about magnetic resonance imaging of the Lisfranc ligament. Secondly, this was an observational trial without control group. Thirdly, the difference between MRI and other examinations remains unknown which need further research.

## Conclusions

On the MRI images, the sagittal section can clearly display the corresponding situation of the Lisfranc joint bone and longitudinal arch of the foot, tolerably display the Lisfranc joint dorsal ligaments and metatarsal ligaments, and poorly display the Lisfranc ligament. The oblique coronal section can clearly display the transverse arch of the foot and clearly display the cross-section of the Lisfranc ligament. The oblique cross-section can clearly display the horizontal arch of the Lisfranc joint and more clearly display its surrounding ligaments and tendons, especially the entire Lisfranc ligament and its attachment points. This is an important section for the diagnosis of Lisfranc ligament injuries. This study provides a certain imaging reference for the MRI scanning, diagnosis, and repair of Lisfranc joint injuries. Further research with large sample size is still needed to confirm the conclusions.

## Abbreviation

MRI: Magnetic resonance imaging

## References

[1] Moore KL (1985). Clinically oriented anatomy.

[2] Horton GA, Olney BW (1993). Deformity correction and arthrodesis of the midfoot with a medial plate. Foot Ankle.

[3] Lapidus PW (1963). Kinesiology and mechanical anatomy of the tarsal joints. Clin Orthop Relat Res.

[4] Jeffreys TE (1963). Lisfranc’s fracture dislocation. A clinical and experimental study of tarsometafarsal dislocations and fracture-dislocations. Bone Joint Surg Br.

[5] Lundberg A, Goldie I, Kalin B, Selvik G (1989). Kinematics of the ankle/foot camplex: plantarflexion and dorsiflexion. Fool Ankle.

[6] Olerud C, Rosendahl Y. Torsion-transmitting properties of the hind foot. Clin Orthop. 1987;(214):285–94. https://www.ncbi.nlm.nih.gov/pubmed/?term=Olerud+C%2C+Rosendahl+Y.+Torsiontransmitting+properties+of+the+hind+foot.+Clin+Orthop.+1987%3B(214)%3A285%E2%80%9394. 3791754

[7] Leenen LP, van der Werken C (1992). Fracture-dislocations of the tarsometatarsal joint, a combined anatomical and computed tomographic study. Injury.

[8] Wanlvenhaus A, Pretterklieber M (1989). First tarsometatarsal joint: anatomical biomechanical study. Foot Ankle.

[9] Sands AK, Grose A (2004). Lisfranc injuries. Injury.

[10] Rosenbaum A, Dellenbaugh S, Dipreta J, Uhl R (2011). Subtle injuries to the Lisfranc joint. Orthopedics.

[11] Desmond EA, Chou LB (2006). Current concepts review: Lisfranc injuries. Foot Ankle Int.

[12] Rankine JJ, Nicholas CM, Wells G (2012). The diagnostic accuracy ofradiographs in Lisfranc injury and the potential value of a cranio-caudal projection. AJR Am J Roentgenol.

[13] Aerts P, Disler DG (1995). Abnormalities of the foot and ankle: MR imaging findings. AJR.

[14] Myerson MS (1989). The diagnosis and treatment of injuries to the Lisfranc joint complex. Orthop Clin North Am.

[15] Hansen ST, Browner BD, Jupiter JB, Levine AM (1992). Tarsometatarsa1(Lisfranc), tarsa1 and intertarsal fracture-dislocations. Skel-etal trauma.

[16] Vuori JP, Aro HT (1993). Lisfranc joint injuries: trauma mechanisms and as-sociated injuries. J Trauma.

[17] Aronow MS (2011). Joint preserving techniques for Lisfranc injury. Techniq Orthopaed.

[18] Thierfelder KM, Gemescu IN, Weber MA (2018). Injuries of ligaments and tendons of foot and ankle: what every radiologist should know. Radiologe.

[19] Tafur M, Rosenberg ZS, Bencardino JT (2017). MR imaging of the midfoot including Chopart and Lisfranc joint complexes. Magn Reson Imaging Clin N Am.

