# Correction to: Magnetic resonance imaging of the Lisfranc ligament

**DOI:** 10.1186/s13018-019-1079-z

**Published:** 2019-02-15

**Authors:** Abulimiti Amuti, Hui-Yong Ding, Li-Guo Liu

**Affiliations:** 1 Department of orthopedics, Second Affiliated Hospital of Xinjiang Medical University, Urumqi, 830000 China; 2 People’s Hospital of Rizhao, Xinjiang Medical University, No. 136 of Tai’an Road, Rizhao, 276800 Shandong Province China

**Correction to: J Orthop Surg Res**

**https://doi.org/10.1186/s13018-018-0968-x**

In the original publication of this article [1], the spelling of the first author's name A.Ablimit was incorrect. The correct name of the author should be Abulimiti Amuti. The original publication has been corrected.
